# A Systematic Review of the Relationship between Familism and Mental Health Outcomes in Latino Population

**DOI:** 10.3389/fpsyg.2016.01632

**Published:** 2016-10-25

**Authors:** Esmeralda Valdivieso-Mora, Casie L. Peet, Mauricio Garnier-Villarreal, Monica Salazar-Villanea, David K. Johnson

**Affiliations:** ^1^Department of Psychology and Public Health, Universidad CentroamericanaAntiguo Cuscatlan, El Salvador; ^2^Gerontology Center, University of KansasLawrence, KS, USA; ^3^College of Nursing, Marquette UniversityMilwaukee, WI, USA; ^4^Instituto de Investigaciones Psicologicas, Universidad de Costa RicaSan Jose, Costa Rica; ^5^Alzheimer's Disease Center, University of Kansas Medical CenterKansas City, MO, USA

**Keywords:** familism, depression, suicide, substance abuse, internalizing, externalizing, Latino

## Abstract

**Background:** *Familismo* or familism is a cultural value frequently seen in Hispanic cultures, in which a higher emphasis is placed on the family unit in terms of respect, support, obligation, and reference. Familism has been implicated as a protective factor against mental health problems and may foster the growth and development of children. This study aims at measuring the size of the relationship between familism and mental health outcomes of depression, suicide, substance abuse, internalizing, and externalizing behaviors.

**Methods:** Thirty-nine studies were systematically reviewed to assess the relationship between familism and mental health outcomes. Data from the studies were comprised and organized into five categories: depression, suicide, internalizing symptoms, externalizing symptoms, and substance use. The Cohen's *d* of each value (dependent variable in comparison to familism) was calculated. Results were weighted based on sample sizes (n) and total effect sizes were then calculated. It was hypothesized that there would be a large effect size in the relationship between familism and depression, suicide, internalizing, and externalizing symptoms and substance use in Hispanics.

**Results:** The meta-analysis showed small effect sizes in the relationship between familism and depression, suicide and internalizing behaviors. And no significant effects for substance abuse and externalizing behaviors.

**Discussion:** The small effects found in this study may be explained by the presence of moderator variables between familism and mental health outcomes (e.g., communication within the family). In addition, variability in the Latino samples and in the measurements used might explain the small and non-significant effects found.

According to the U.S. Census Bureau (2011), the Latino population has increased by almost 43% in 10 years (2000–2010) within the U.S. This growth accounts for over half of the increase in the total U.S. population (U.S. Census Bureau, 2011). Along with the rapid increase of this population, the magnitude of their socio-economic and health indicators have become public interest: 22% of Latino adults live below the poverty level (U.S. Census Bureau, 2013); 37% of Latinos are uninsured (Brown et al., 2000), only 1 out of 11 Latino-Americans with mental disorders contact a mental health care specialist (U.S. Department of Health and Human Services, 2001), and Latino adolescents have high rates of major depressive episodes and a low percentage is receiving treatment [Substance Abuse and Mental Health Services Administration (SAMHSA), 2015]. Nevertheless, conflicting evidence in mental health outcomes is found when the adult Latino population is analyzed. Alegría et al. (2008) found that Latino adults have lower prevalence of several mental health disorders compared to non-Latino whites. This type of evidence gives support to the Hispanic or Immigrant Paradox that has become a matter of interest in the past 10 years. This paradox establishes that Latinos or Hispanics sustain a health advantage over non-Hispanic whites (Elo et al., 2004; see Blanco et al., 2013). In this study, we considered Latinos or Hispanic as exchangeable words, although they do not refer to the same concept (see Gonzalez, 1992, November 15).

Even though the Hispanic paradox places immigrant Hispanics in a healthier position than non-Hispanic whites and Hispanic-Americans, the mental health outcomes reported by the American Psychiatric Association (APA, 2014); Substance Abuse and Mental Health Services Administration (SAMHSA, 2015), and the National Alliance for Mental Illnesses (NAMI, 2002) indicate that Latinos in general are at risk for several conditions. Both evidences for and against the Hispanic Paradox have placed a demand for research, to understand the factors associated with the health outcomes of Latinos. Hispanic cultural values have become an area of interest due to the suggested protective factor that they may serve (Smokowski et al., 2009; Calzada et al., 2012). One of the most studied Hispanic core cultural values is *familismo* or familism in which a higher emphasis is placed on the family unit in terms of respect, support, obligation, and reference (Calzada et al., 2012). It is of interest in this systematic review to analyze the relationship between familism and mental health outcomes in Latino populations.

## Familism

Familism refers to the cultural value that one's family is expected to provide necessary emotional and instrumental social support when needed (Sabogal et al., 1987; Calzada et al., 2012). At the same time, familism creates a sense of obligation to take care of one's family, and to take one's family into consideration when making decisions. In this sense, family becomes a source of information for behaviors and attitudes (Parsai et al., 2009; Davila et al., 2011). There are three measures (structural, behavioral, and attitudinal) that can be used to assess levels of familism. Structural familism is the physical proximity to family members, behavioral is the behavior in relation to their family's values and expectations and lastly is attitudinal familism. Attitudinal familism measures an individual's thoughts and feelings on the three different aspects of familism: (a) supportive familism signifies the level to which the individual feels supported by his or her family and the degree of closeness in their family; (b) obligatory familism is the extent to which an individual believes the family has a responsibility to provide support (economic, social, or emotional) to other family members; and (c) referent familism is the extent to which one maintains behaviors that are consistent with the family values and expectations (Sabogal et al., 1987; Marsiglia et al., 2009). It is frequently specified that involvement in Latino culture is critical for high familism attitudes and that greater acculturation to American culture and more time spent in the United States is linked to lower levels of familism (Smokowski et al., 2009). Studies included in this meta-analysis measured attitudinal familism, through a series of questions or familistic statements based on a Likert scale.

### Familism and mental health outcomes

Attitudinal familism has been implicated by many as a protective variable against mental health problems and fosters the growth and development of children (Zeiders et al., 2013). There is substantial literature to suggest this is a protective factor particularly among Latinos and also among Asians (Calzada et al., 2012).

It has been found that the high levels of family supportiveness among Mexican American families serve as a protective factor during times of crises and psychological distress (Umaña-Taylor et al., 2011). Furthermore, poor mental health has been linked to low levels of familism (Ornelas and Perreira, 2011). In a 2011 study, familism was found to moderate some variables of stress (i.e., acculturative stress) but was found insignificant in others (i.e., discrimination, economic hardship; Umaña-Taylor et al., 2011). The relationship between familism and depression and other internalizing symptoms varies throughout the literature with some studies showing no interaction between the two, others showing more depressive symptoms with higher familism levels (Zeiders et al., 2013) and some displaying familism as a protective buffer against depression (Ornelas and Perreira, 2011). These inconsistencies require further research between familism and depression.

Research investigating the association between familism and suicidal attempts has suggested familism as a protective factor because the greater sense of loyalty to the family offers a reason to live (Garza and Pettit, 2010). However, there is contradicting literature; some researchers have found that many adolescent Latina suicide attempters take on blame and guilt for family problems suggesting that perhaps familism could create an additional responsibility upon young Latinas (Kuhlberg et al., 2010). This is a very under researched area that necessitates more attention.

There is a plethora of consistent research on lower substance use, binge drinking, and smoking in Hispanics implying that cultural values play a significant role. A study done at the Institute for Health Promotion and Disease Prevention stated that perhaps the relationship that continues to appear between lower levels of substance use and Hispanics is due to familism by enhancing the responsibilities of the individual to positively represent their family (Soto et al., 2011). Many studies strictly look at drinking while others look at illicit drug use or marijuana, causing a need for more comprehensive research.

As previously stated, the relationship between familism and internalizing symptoms is inconsistent throughout the literature. There have been studies showing that familism moderates the relationship between risk factors (i.e., parent adolescent conflict) and outcome (i.e., internalizing symptoms), while others show contradicting evidence (Vargas et al., 2013). One study by Smokowski and colleagues in 2009 supports familism as a protective cultural factor against internalizing symptoms, noting that higher familism is related to lower internalizing symptoms (Smokowski et al., 2010). Other studies claim the protection of familism is mediated by parent-adolescent conflict (Smokowski and Bacallao, 2007; Smokowski et al., 2007). While there is much literature in support of familism as a protective factor, there is still inconsistency, demanding more research before conclusions can be confidently drawn.

Protective effects of familism on externalizing behaviors have been theorized to be because adolescents with higher familism attitudes feel more obligation toward the family unit and see acting out as disgracing their family (Germán et al., 2008). In particular, studies have found familism to be protective against deviant behavior despite being exposed to deviant peers (Germán et al., 2008). Another study found that in addition to mediating the risk factor of deviant peer association, higher familism values resulted in lower associations with deviant peers (Roosa et al., 2011). While the literature is fairly consistent, it is also highly restricted to child participants requiring more research across a larger age span (Roosa et al., 2011).

## Aims and hypothesis

The familism component has been implicated as a potential protective factor within these mental health outcomes, nevertheless the empirical evidence is somewhat conflicting. To solve this, the present systematic revision aims at assessing the relationship between familism and five mental health outcomes: depression, suicide, substance abuse, internalizing, and externalizing behaviors. The methodology selected to pursue this aim is a meta-analysis, which is a type of research conducted in order to piece together various published studies and pieces of literature in an attempt to find patterns and implications that may otherwise go overlooked. We hypothesize that there will be a large effect size in the relationship between familism and depression, suicide, substance abuse, internalizing, and externalizing behaviors in the Latino or Hispanic population within the United States. A large effect size would suggest that familism serves as a protective factor for the mental health outcomes of interest in this study.

## Methods

PsycINFO database was systematically searched to identify the research articles used in this systematic review. The article selection process was conducted using the four-step process suggested by the PRISMA group (Moher et al., 2009) for systematic reviews. From the original search, 141 articles were identified (see Table 1). Researchers reviewed titles and abstracts of the articles to determine if the studies were conducted with Latino population. During the screening step, those articles that were (a) duplicated records, (b) expert opinion, (c) literature reviews, and (d) qualitative studies were excluded. The result was 54 potential articles to be examined further for eligibility and inclusion. The criteria used for review of articles in the two final steps are described in Table 1. The number of studies included in the analysis was of 39 published articles and dissertations.

**Table 1:** **Article selection process using PRISMA (2009) Steps**.

**Step**	**Criteria**	**No. of studies that fulfilled criteria**
Identification	1. Terms included in search:	141
	(a) familism and depression, (b) familism and suicide, (c) familism and internalizing, (d) familism and substance, and (e) familism and externalizing.	
	2. Publication year range: 2005–2015.	
Screening	Titles and abstracts contained participants from Latino or Hispanic populations, born in or outside the U.S. Duplicated records, expert opinions, literature review, and qualitative studies were removed.	54
Eligibility	Full-text articles were quantitative studies with a measure of attitudinal familism and one of the mental health outcome variables of interest.	50
Included	Full-text articles had:	39
	1. Defined sample size of Latino or Hispanic population. 2. Clearly indicated the instrument used to measure familism and the mental health outcome variable of interest; 3. Reported statistics of the direct relationship between familism and, at least, one of the other mental health outcome variables of interest; and 4. Based the analysis on original data collection, not replicated data.	

Researchers examined each study for the following: (a) population, (b) sample size, (c) sample characteristics, (d) measure of familism used, (e) mental health outcome analyzed, (f) measure of mental health outcome used, and (g) type of statistical analysis used. This information is presented in Table 2 for each of the 39 articles.

**Table 2:** **Studies included in meta-analysis**.

**Study**	**Sample**	**Familism measure**	**Mental health outcome**	**Instrument used**	**Statistics for analysis**	**Cohen's *d***
Baham, 2009	119 Mexican-American Adolescents, Age range: 15–17 years	MACVS	Substance abuse	YRBS	Correlation coefficients	0.1202
			Internalizing	RCMAS and CDI		0.1605
			Externalizing	BPI		0.1403
Baumann et al., 2010	169 Latina adolescents, Mean age: 15.19 years Women only, 73% born in the U.S.	AFS	Suicide	YSR	M and SD	0.2453
			Internalizing	YSR	Correlation coefficients	0.7903
			Externalizing	YSR		0.1909
Burrow-Sanchez et al., 2014	106 Latino adolescents, Mean age: 15.30 years, Male: 91.5%	AFS	Depression	BDI-II	Correlation coefficient	0.6198
	Born in the U.S.: 63.2%					
Campos et al., 2014	173 Latino, Mean age: 19.93 years, Born in the U.S.: 80%	AFS	Depression	CES-D	Means and standard deviations^a^	0.1202 Obligational
						0.0801 Supportive
						0.0600 Reference
Cavanaugh, 2015	133 Latino adolescents, Mean age: 12.88 years, Female: 51%	MACVS	Internalizing	YSR	Correlation coefficients for both variables with each form of familism	0.2213 Obligational
						0.2828 Supportive
						0.2828 Reference
			Externalizing			0.4727 Obligational
						0.4082 Supportive
						0.3242 Reference
Chavez-Korell et al., 2014	98 Latino older adults, Mean age: 71.04 years, Female: 66%	PHFS	Depression	PHQ-9	Correlation coefficient	0.4945
Cupito et al., 2015	179 Latino adolescents, Mean age: 14 years, Female: 52%	AFS	Depression	MFQ	Correlation coefficients for males and females, separately	0.6060 Females
						0.4727 Males
De Santis et al., 2012	46 Hispanic male with HIV infection, Mean age: 44.2 years, Male only	AFS	Depression	CES-D	Correlation coefficient	0.2173
Diaz, 2011	194 Mexican-American adolescents, Mean age: 12.88 years, Female: 52.6%	MACVS	Internalizing	RCMAS and CDI	Correlation coefficients	0.4511
			Externalizing	BPI		0.020
			Substance abuse	YRBS		0.2213
Fallah, 2014	170 Latino adolescents, Mean age: 14.06 years, Female: 55%, Born outside the U.S.: 67%	AFS	Depression	MFQ	Correlation coefficient	0.4727
Garza and Pettit, 2010	61 Mexican or Mexican-American, Mean age: 34.55 years, Women only, All born outside the U.S.	AFS	Depression	BDI-II	Correlation coefficients	0.3450
			Suicide	INQ		0.0400
Germán et al., 2008	598 Mexican-origin adolescents, Born in the U.S.: 79.1%	MACVS	Externalizing	CBCL TRF	Correlation coefficients	0.1001 (Mother report)
						0.0200 (Father report)
						0.1403 (Teacher 1 report)
						0.2213 (Teacher 2 report)
Howarter, 2014	1386 Latino adolescents from project RED, Age range: 12–16 years, Female: 53.5%	Four-item scale	Depression	CES-D	Correlation coefficients	0.1807 Females
			Substance abuse	YRBS	for women and men separately, of depression, smoking and alcohol	
						0.3871 Males
						0.1605 Smoking females
						0.1403 Smoking males
						0.1001 Alcohol females
						0.1403 Alcohol males
Keeler et al., 2014	84 Mexican-American, Mean age: 30 years, Female: 48%, Born in the U.S.: 36%	Six-item scale	Depression	BDI-II	Correlation coefficients	0.7473
Kissinger et al., 2013	91 Latino migrant workers, Male only	AFS	Substance abuse	NSDUH tool, AUDIT	M and SD for each group	0.4145 NSDUH
						0.1440 AUDIT
Kuhlberg et al., 2010	226 Latina adolescents, Mean age: 15.47 years, Women only	AFS	Suicide	Suicide Attempt	Mean and standard deviation Correlation coefficient	0.2168
			Internalizing	YSR		0.1403
Kuo et al., 2015	246 Latino adolescents, Mean age: 17.72 years, Female: 51%	MACVS	Depression	CED-S	Correlation coefficients for time 1	0.0801 (Mothers)
						0.1001 (Fathers)
Lac et al., 2011	1369 Latino adolescents, Mean age: 13.99 years, Female: 54.3%	Four-item scale	Substance abuse	One-item scale on marijuana use	Correlation for time 1 only	0.1605
Lin, 2007	596 Mexican-American adolescents, Mean age: 12.31 years, Female: 51%	MACVS	Substance abuse	YRBS	Correlation coefficient	1.2904
Losada et al., 2006	48 Adult caregivers, Mean age: 58.0 years, Female: 79.2%, Born in US: 54%	AFS	Depression	CES-D	Correlation coefficient	0.3871
Muñoz-Laboy et al., 2014	259 Formerly incarcerated Latino Men	AFS	Depression	BSI	Odds ratio	0.0168
Ocegueda, 2009	95 Mexican-American adolescents, Age range: 18–19 years, Female: 63.2%	Seven-item scale	Externalizing	Self-Report Delinquency Scale	Correlation	1.0276
Ornelas and Perreira, 2011	281 First-generation Latino youth parents, Mean age: 40 years, Female: 84%	Seven-item scale	Depression	PHQ-9	Odds ratio of familism and each depression measurement	0.0342
				CES-D		0.1096
Peña et al., 2011	216 Adolescent Latinas reporting (suicide attempt vs. no suicide attempt), Mean age: 15.5 years, Women only	AFS	Suicide	Suicidal attempt	Mean and standard deviations of familism for each group (attempters vs. non-attempters)	0.1219
Reid-Quiñones, 2011	222 Latino emerging adults, Age range: 18–25 years, 61.7% Female, 72.5% born in the U.S.	MACVS	Substance abuse	CADS	Odds ratio for cigarette, marijuana and alcohol consumption	0.000 Cigarette
				DAST-20		0.0217 Alcohol
						0.0109 Marijuana
Roosa et al., 2011	750 Mexican-origin adolescents, Mean age: 10.4 years, Female: 48.7%	MACVS	Externalizing	DISC	Correlation coefficient	0.1403
Soto et al., 2011	1616 Hispanic students, Age range: 13–15 years, Female: 54%, 88% born in the U.S.	Five-item scale	Substance abuse	YRBS	Odds ratio for marijuana, alcohol and cigarette use	0.0476 Cigarette
						0.0527 Alcohol
						0.0724 Marijuana
Smokowski and Bacallao, 2007	323 Latino adolescents, Mean age: 15 years, Female: 51%, 97% born outside the U.S.	Six-item scale	Internalizing	YSR	Correlation coefficient	0.7791
Smokowski et al., 2007	100 Latino adolescents, Mean age: 15 years, Female: 54%, All born outside the U.S.	Seven-item scale	Internalizing Externalizing	YSR	Correlation coefficient	0.6190 0.3367
Smokowski et al., 2009	288, Latinos adolescents, Mean age: 15 years, Female: 54.5%, 67% born outside the U.S.	Seven-item scale	Internalizing	YSR	Correlation coefficient	0.6755
Smokowski et al., 2010	349 Latinos adolescents, Mean age, not reported, 66% born outside the U.S.	Seven-item scale	Internalizing	CBCL, YSR	Correlation coefficient	0.5833
Stein et al., 2015	173 Latino adolescents, Mean age: 14.08 years, Female: 53.8%, Mexican origin: 78%	AFS	Depression	MFQ	Correlation coefficient	0.4082
Telzer, 2012	385 Latino adolescents, Mean age: 15.01 years, Female: 51%	Twelve-item scale	Substance abuse	YRBS	Correlation coefficients	0.3660
Umaña-Taylor et al., 2011	207 Mexican-origin adolescents mothers, Mean age: 16.23 years, 64.6% born in the U.S.	MACVS	Depression	CES-D	Correlation between familism and depression	0.1605
Vargas et al., 2013	750 Mexican-American, Age range: 10–12 years, Female: 49%, 70% born in the U.S.	MACVS	Internalizing	DISC-IV	Correlation coefficients	0.0200
			Externalizing			0.0400
Venegas et al., 2012	160 Hispanics, Mean age: 19.9 years, Female: 50%	AFS	Substance abuse	DDQ	Mean and standard deviations	0.0446
Zapata et al., 2016	102 Latino adolescents, Mean age: 13.8 years, Male: 52.9%, Born in the U.S.: 51%	Seven-item scale	Substance abuse	Use of alcohol, cigarette and marijuana in the last 30 days	Correlation coefficients	0.5608 Cigarette
						0.3450 Alcohol
						0.5833 Marijuana
Zayas et al., 2009	140 Latinas adolescents, Mean age: 15.21 years, Women only, 72.14% born in the U.S.	AFS	Suicide	Suicidal attempt	Means and standard deviations	0.3106
Zeiders et al., 2013	492 Latino adolescents, Age range: 13–23 years, Born in the US: 58%	MACVS	Depression	CES-D	Regression coefficients for each type of familism subscale	0.1106 Obligational
						0.1711 Supportive
						0.1675 Reference

*AFS, Attitudinal Familism Scale; MACVS, Mexican-American Cultural Values Scale; CES-D, Center for Epidemiological Studies Depression Scale; PHQ-9, Patient Health Questionnaire-9; YSR, Youth Self-Report; CBCL, Child Behavior Check List; BDI-II, Beck Depression Inventory-II; INQ, Interpersonal Needs Questionnaire; DISC-IV, Diagnostic Interview Schedule for Children; YRBS, Youth Risk Behavior Survey; RCMAS, Revised Children's Manifest Anxiety Scale; CDI, Children Depression Inventory; DDQ, Daily Drinking Questionnaire; BPI, Behavior Problems Index; NSDUSH tool, National Survey on Drug Use and Health tool; AUDIT, Alcohol Use Disorders Identification Test; CADS, Core Alcohol and Drug Survey; DAST-20, Drug Use Screening Test-20; TRF, Teacher Report Form; MFQ, Mood and Feelings Questionnaire; BSI, Brief Symptoms Inventory for Depression*.

a *With the provided data from the SEM we simulated data a 1000 times and estimated the correlations between the variables of interest. We used the mean and 95% CI correlations for the simulations*.

### Studies samples

All studies contained a sample of Latinos or Hispanics living in the United States. The studies required participants to self-identify as Hispanic or Latino. Participants were either foreign born or U.S. born. Those participants who were born in the U.S. had a Latin American background (e.g., one or both parents were born in Mexico, Central, or South America). As presented in Table 2, many of the studies contained samples of strictly adolescents. Only seven studies had adult participants (e.g., mean age higher than 18 years old). In terms of gender distribution, three studies were conducted with only a male population, and six studies with a female population exclusively. The rest of studies had both male and female Latinos in their samples. Most participants belonged to middle-, and high-school communities. Other participants were defined by one of the following different characteristics: adult caregivers, adolescent mothers, persons with HIV infections, formerly incarcerated Latinos, specific intervention-group participants (e.g., suicide attempter), or prevention/research group participants (e.g., RED, CAMINOS).

### Measures

#### Familism

A variety of scales were used to determine familism scores for the participants in each study. The most widely used was the Attitudinal Familism Scale (AFS) generated by Lugo Steidel and Contreras (2003), which was used in 17 out of 39 studies. The following most used scale was the Mexican American Cultural Values Scale (MACVS; Knight et al., 2009), which was used in 11 out of the 39 studies. The rest of the studies used a scale from the University of California—San Francisco (Marin et al., 1987; Sabogal et al., 1987) or generated their own individual 3–7 item Likert scales.

#### Depression

The most used measure was the Center for Epidemiologic Studies Depression Scale, CES-D (Radloff, 1977; Santor and Coyne, 1997), utilized in eight studies. The Beck Depression Inventory II (Beck and Steer, 1991; Steer et al., 1998) was the second most used instrument for depression, present in three studies. Other studies used the Mood and Feelings Questionnaire by Angold et al. (1995), the Patient Health Quesitonnaire-9 (Kroenke and Spitzer, 2002), the Depression Brief Symptom Inventory, BSI (Derogatis, 1993), and the Child Depression Inventory (Kovacs, 1992).

#### Suicide

Suicidal attempts in each study were defined as any act of self-harm or intention of hurting or killing oneself. There was no scale to measure suicide; in each of the four suicidal studies, the participants were chosen for each condition based on medical records and/or reports by the school or family. Suicidal attempts, regardless of the level of lethality, were considered under the “suicide” condition while suicidal ideation was considered under internalizing.

#### Substance use

Substance use was self-reported by the participants. A variety of substances were encompassed in the studies including marijuana, alcohol, illicit drugs, and cigarettes. The most used instrument for self-reporting substance was the Youth Risk Behavior Survey (Centers for Disease Control and Prevention (CDC), 2000) in six of the studies. Other studies administered the Daily Drinking Questionnaire (Venegas et al., 2012), Alcohol Use Disorders Identification Test AUDIT (Cherpitel and Bazargan, 2003), the Core Alcohol and Drug Survey CADS (Presley et al., 1998), the Drug Abuse Screening Test DAST-20 (Skinner, 1982), and a self-generated report of substance abuse in the last 30 days.

#### Internalizing and externalizing symptoms

The most frequently used scale to measure internalizing and externalizing symptoms in participants was the Youth Self Report Scale YSRS (Baham, 2009), in six out of 39 studies. Also used was the Child Behavior Checklist (Achenbach, 1991), Revised Adolescent's Manifest Anxiety Scale RCAMS (Reynolds and Paget, 1981), the Diagnostic Interview Schedule for Children DISC-IV (Shaffer et al., 2000), Behavior Problems Index (adapted from the YSRS; Baham, 2009), Teacher's Report Form (Achenbach and Rescorla, 2001), and a Self-Report Delinquency Scale.

### Data analysis

The studies included in this review contained various types of analyses, yielding to numerically different values. Studies presented one of the following data: Pearson's *r* correlation coefficient, odds ratios, hierarchical, standardized and unstandardized regression coefficients, or means and standard deviations for group comparisons (e.g., suicide attempter vs. non-attempter). In order to calculate a summarized effect of these studies, the data extracted from the articles were converted to standardized effect size measures. For each study, an effect size was calculated with Cohen's *d* (see Borenstein et al., 2009). The following formula was used for calculating Cohen's *d* of Pearson's *r* correlation coefficient:

(1) $$\begin{array}{l} d=\frac{2\text{r}}{1-r^{2}} \end{array}$$

For mean scores:

(2) $$\begin{array}{l} d=\frac{{|\bar{x}}_{1}\;-\;{\bar{x}}_{2}|}{\sqrt{\frac{S_{1}^{2}\;+\;S_{2}^{2}}{2}}} \end{array}$$

For odds ratios:

(3) $$\begin{array}{l} d=\frac{\sqrt{3}log\left(OR\right)}{\pi } \end{array}$$

And, for regression coefficients:

(4) $$\begin{array}{l} \frac{B}{\left(SD_{1}/SD_{2}\right)} \end{array}$$

Total effect sizes were calculated for each mental health outcome variable (e.g., depression, suicide, internalizing behaviors, substance abuse, externalizing behaviors). Results were weighted based on sample sizes (*n*):

(5) Σ(n1 * Cohen's D1)+…+(nx * Cohen's Dx)Σn

The results presented in the following section are organized into five categories: depression, suicide, substance abuse, internalizing and externalizing behaviors.

## Results

**Figure 1:**
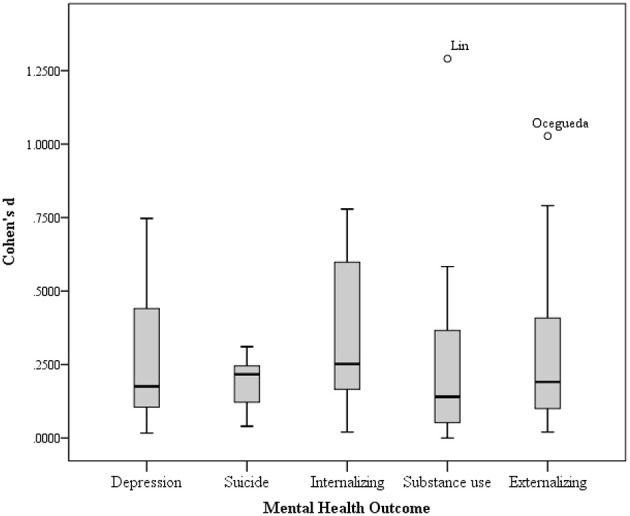
**Effect sizes (Cohen's *d*) per mental health outcome variable**.

Effect sizes for Cohen's *d* were categorized as small, medium, or large, using the 0.2, 0.5, and 0.8 benchmarks, respectively (Borenstein et al., 2009). As Figure 1 shows, of the five dependent variables, internalizing behaviors (*d* = 0.33), suicide (*d* = 0.20), and depression (*d* = 0.21) showed a small effect with familism. The remaining two variables did not display a significant relationship.

As Table 2 indicates, individual studies showed stronger associations between familism and the mental health outcomes analyzed than the overall effect sizes reported above. The effect sizes for depression ranged from *d* = 0.02 to *d* = 0.75. Out of 24 studies on depression, 13 showed no significant effect (54.2%), 8 showed a small effect (33.3%), and 3 had moderate effect sizes (12.5%).

Substance use analysis included 21 studies, with effect sizes ranging from *d* = 0.00 to *d* = 1.29. Fourteen studies showed no significant effect sizes (66.7%); 4 studies had a small effect (19.0%) and 3 showed moderate effect sizes (14.3%).

The range of effect sizes for suicide was narrower, suggesting less variability within the relationship of familism and suicide (range of *d* [0.04, 0.31]). Out of 5 studies on suicide, 3 had small effect sizes and the rest no significant effects.

For the internalizing behaviors analysis, the effect sizes were distributed similarly. With a range of *d* = 0.02 to *d* = 0.78, there were 4 internalizing studies in each category: moderate, small and non-significant effect (33.3% each). With a very different distribution of effect sizes, the externalizing behaviors analysis showed a range of *d* = 0.02 to *d* = 1.03. Half of the studies showed no significant effects (7 out of 14 studies), 5 had small effect sizes, and only 2 reached moderate sizes for the effect.

## Discussion

The results of this meta-analysis showed a small effect of familism on depression, suicide, and internalizing behaviors, and no effect for substance use and externalizing symptoms.

The small effects on depression, suicide and internalizing behaviors are coherent since depression and suicidal behavior are frequently associated with internalizing symptoms (depression, anxiety, mood disorders; Bridge et al., 2005). An important part of the etiology of mood disorders and other internalizing disorders, involve reduced or inadequate family support (Sheeber et al., 1997; Fristad et al., 2003). Therefore, it would make sense that attitudinal familism, which includes the feeling of support by one's family, would be related with lower rates of internalizing symptoms and consequently, lower suicide rates and depression symptoms. Family support reflects a positive family functioning which serves as a buffer effect for stressful life experiences in childhood, adolescence, and adulthood (Tubman and Windle, 1995). Since Latino adolescents are considered at a higher risk of suicide [Substance Abuse and Mental Health Services Administration (SAMHSA), 2015], the summary effect found in the present study is suggesting that many adolescents refrain from turning to extreme acts such as suicide, by reaching out to family members when support is needed (Dunham, 2004). With low levels of familism values it is likely that an adolescent struggling with stressful life events or internalizing symptoms would be hesitant to turn to parents and family members for support, subsequently increasing the risk and result of depression and suicidal behaviors. Nevertheless, the effect sizes for depression, suicide and internalizing behaviors should be interpreted with caution. The effect sizes for the three variables were small. That is, the results are suggesting that attitudinal familism has a real effect on these variables, but it can only account for a small proportion of variance for depression, suicide and internalizing symptoms. Potential reasons for the small effect are address at the end of this section.

The results of the present study showed no significant effect sizes of familism on substance use and externalizing behaviors. Although the magnitude of the effects is indicating that family factors alone play only a minimal role in causing or predicting externalizing symptoms and substance abuse, one's perception of familial support is critical in the causes of substance use and externalizing psychopathologies (Beitchman et al., 2005). Then, how can the non-significant effect size be explained in Latino population? Maybe, attitudinal familism as a cultural value is not as important as behavioral familism. That is, manifest behaviors of support and good communication. Kam and Yang (2014) found that attitudinal familism was not significantly related to personal substance-abuse norms in adolescents Latinos, but targeted mother-child communication was. This finding could be particularly relevant for Latino adolescents born in the U.S., whose levels of acculturation may be higher and cultural familism lower in comparison to those born and raised outside the U.S. (see Cervantes, 2002). Recent findings support that both U.S.-born and foreign-born Latinos experience acculturation stress, even when they are completely bilingual and bicultural (Cervantes et al., 2013). Acculturation stress has been found positively associated with externalizing problems or conduct problems in both populations. Such relationship is moderated by parental monitoring (i.e., behavioral familism), which provides a protective effect against externalizing problems (Hurwich-Reiss and Gudiño, 2015).

The small and non-significant effects found in this meta-analysis could be the result of limitations from the studies included in this meta-analysis, as well as from our methods. Limitations on measurement variability and sample variability should be noted and improved upon in future research.

The first concern arises from the measurements used in the studies included in the meta-analysis. As reported on Table 2, studies measured familism with the MACVS, the Familism Scale of Sabogal, and the Familism Scale of Lugo. Other studies just built their own Likert scale for familism, using 4–7 items. Not all of the studies differentiated the subscales scores of familism (supportive, obligatory, and referent familism). And those studies which built their own instruments did not mention a particular type of attitudinal familism, addressing the variable in general terms. The differences in the measurement raises concern because there is evidence that supportive and referent familism is a protective factor for mental health outcomes, while obligatory familism is not (Zeiders et al., 2013). Therefore, those studies measuring the subtypes of familism that averaged across the scale could have larger effect sizes if they were analyzed separately. And, the rest of studies that did not differentiate between the subtypes may have somewhat ambiguous conclusions.

In terms of sample variability, it is important to address differences within Latino population. Social psychology has explained in a simple and easy way how humans use heuristics for categorizing persons in groups and assigning equal values and characteristics to them. A phenomenon called the out-group homogeneity effect, which simplifies our thinking processes (Quattrone and Jones, 1980). This effect is relevant to interpret the meta-analysis because the studies included had relevant within-group differences in terms of how their samples were conceptualized and characterized. From those studies that reported specific information of their sample, most had Latino participants who were born in the U.S. (ranging from 36 to 100% participants born in the U.S., see Table 2). Few studies just requested participants to self-identify as Latino or Hispanic. No information of country of origin or numbers of years in the U.S. was reported. Although, all participants had a Latin American background, they all cannot be considered equal or homogenous. That is, the out-group homogeneity effect should be avoided for two reasons. In the first place, the Hispanic paradox evidences differences between Hispanic immigrants and U.S. born persons of Hispanic origin, in terms of health (Blanco et al., 2013), which suggests a cultural protective factor that is diminished by acculturation (Calzada et al., 2012). In addition, when analyzing results from Substance Abuse and Mental Health Services Administration (SAMHSA, 2015) and Alegría et al. (2008), differences between adults and adolescents Latinos arise, with adolescents being at greater risk of mental health disorders. The difference between age groups could be explained using the “dual frame of reference” by Suárez-Orozco and Suárez-Orozco (1995), which explains how Latino immigrants use their families back home as a reference when reflecting about their lives in the U.S. while U.S. born Latinos could be comparing themselves to American peers. Since social and economic conditions of the families back home are often worse, the U.S. immigrants are less likely to experience distress, in comparison to U.S. born Latinos (U.S. Department of Health and Human Services, 2001). And in second place, and equally important, Latinos are usually defined as individuals who speak Spanish and have a Latin American background; and in consequence persons from more than 15 different countries are grouped into one sole category (using again the out-group homogeneity effect). Within-group variability in Latino population is particularly important when studying immigrants to the U.S. The U.S. Department of Health and Human Services (2001) gives reasons to care about the differences within Latino population. The reasons that motivate a Mexican origin person to migrate to the U.S. is different to those that motivate a Central American or a South American. According to the report, Mexicans are pushed by the economic hardship, while Central American were mostly driven by the civil wars and violent conflicts. South Americans who have better economic conditions and less violence, are probably motivated to migrate for other reasons. This difference within group should be addressed in future studies, because depression, alcoholism, and PTSD may be at higher risk in Central American immigrants than in other immigrants of the region.

Therefore, studies including all U.S.-born and foreign-born Latinos in one group are not attending important within group differences. Berdahl and Torres Stone (2009) conducted a study that enlightens such differences. They compared Mexicans, Cubans, and Puerto Ricans to non-Latino whites and found that Mexicans were less likely to use mental health services, compared to all previous groups. The researchers discuss their findings by addressing the diverse sociopolitical relationships of these countries with the U.S.

Future meta-analysis on familism and mental health outcomes should take into account the limitations in the present study. First, this study was limited to attitudinal familism. Therefore, the current results cannot be generalized to all types of familism. We consider that separate analysis should be conducted for each type of familism, including behavioral or manifest acts of familism. Separate analysis would help understand the differential effect of each type of familism on each type of mental health outcomes in Latinos. Second, the measure of each construct widely varied between studies. To protect against potential method variability affecting the results, future meta-analysis should consider including studies with similar measurement scales. Third, the study did not limit the sample by age, country of origin, time in the U.S. or socioeconomic status. With a larger body of publications on this topic a more stringent inclusion criteria should be used. To reduce sample variability, future researchers should to opt for a specific age range, geographical area (e.g., Mexican, Central American, Caribbean, or South American origin) and time in the U.S. The concerns about sample variability have been exposed above.

The interest of familism in Hispanic or Latino cultures is a relatively new but quickly growing field of interest. Further research and evidence proving the effects of familism on mental health could give a greater grasp on etiology and treatment options for psychopathologies. The aspects discussed in this section in terms of sample characteristics and measurement should be taken into consideration for future studies.

## Author contributions

EV and CP conducted the literature research and conducted the statistical analysis. CP wrote most of the Introduction and EV wrote most of the Methods, Results and Discussion sections. MG helped with data analysis and interpretation. MS and DJ supervised the study and gave feedback, made corrections and contributed with important ideas for the introduction and discussion.

## Funding

Support from NIA Developmental Grant R21TW009665 and the University of Kansas Open Access Author Fund.

### Conflict of interest statement

The authors declare that the research was conducted in the absence of any commercial or financial relationships that could be construed as a potential conflict of interest.
